# Discrimination of conventional and organic white cabbage from a long-term field trial study using untargeted LC-MS-based metabolomics

**DOI:** 10.1007/s00216-014-7704-0

**Published:** 2014-03-12

**Authors:** Axel Mie, Kristian Holst Laursen, K. Magnus Åberg, Jenny Forshed, Anna Lindahl, Kristian Thorup-Kristensen, Marie Olsson, Pia Knuthsen, Erik Huusfeldt Larsen, Søren Husted

**Affiliations:** 1 Department of Clinical Science and Education, Karolinska Institutet, Södersjukhuset, 11883 Stockholm, Sweden; 2 Plant and Soil Science Section, Department of Plant and Environmental Sciences, Faculty of Science, University of Copenhagen, Thorvaldsensvej 40, 1871 Frederiksberg C, Denmark; 3 Department of Analytical Chemistry, Stockholm University, Svante Arrhenius väg 16C, 10691 Stockholm, Sweden; 4 Department of Oncology-Pathology, Science for Life Laboratory, Clinical Proteomics and Mass Spectrometry, Karolinska Institutet Science Park, PO Box 1031, 171 21 Solna, Sweden; 5 Faculty of Science and Technology, Department of Food Science, Århus University, Kirstinebjergvej 10, 5792 Årslev, Denmark; 6 Department of Horticulture, Swedish Agricultural University, PO Box 103, 230 53 Alnarp, Sweden; 7 National Food Institute, Technical University of Denmark, Mørkhøj Bygade 19, 2860 Søborg, Denmark; 8 Present Address: Plant and Soil Science Section, Department of Plant and Environmental Sciences, Faculty of Science, University of Copenhagen, Højbakkegårds Alle 13, 2630 Tåstrup, Denmark

**Keywords:** Conventional agriculture, Long-term field trial, Metabolomics, Organic agriculture, White cabbage

## Abstract

The influence of organic and conventional farming practices on the content of single nutrients in plants is disputed in the scientific literature. Here, large-scale untargeted LC-MS-based metabolomics was used to compare the composition of white cabbage from organic and conventional agriculture, measuring 1,600 compounds. Cabbage was sampled in 2 years from one conventional and two organic farming systems in a rigidly controlled long-term field trial in Denmark. Using Orthogonal Projection to Latent Structures–Discriminant Analysis (OPLS-DA), we found that the production system leaves a significant (*p* = 0.013) imprint in the white cabbage metabolome that is retained between production years. We externally validated this finding by predicting the production system of samples from one year using a classification model built on samples from the other year, with a correct classification in 83 % of cases. Thus, it was concluded that the investigated conventional and organic management practices have a systematic impact on the metabolome of white cabbage. This emphasizes the potential of untargeted metabolomics for authenticity testing of organic plant products.

**Electronic supplementary material:**

The online version of this article (doi:10.1007/s00216-014-7704-0) contains supplementary material, which is available to authorized users.

## Introduction

Organic agriculture, in contrast to its conventional counterpart, does by regulation not make use of synthetically produced fertilizers and pesticides. Instead, organic farming employs different crop rotations and use of moderate amounts of animal and green manures for nutrient supply and disease-resistant varieties, among other approaches, for pest control.

While it is evident that on average, organic products contain less pesticide residues, it is disputed to which extent different farming practices lead to systematic differences in the endogenous chemical composition of a crop. Numerous studies have compared the nutrient content of crops from organic and conventional agriculture. Recent reviews attempting a formal meta-analysis [1, 2] have come to different conclusions as to whether or not the production system has a substantial impact on crop nutrient content or quality. These reviews are largely based on the same original studies, and it appears to be a matter of the applied statistics, whether many [1] or few [2] significant (micro)nutrient differences are detected between organic and conventional crops.

As an exception to this inconsistency, recent reviews have consistently found significantly higher phosphorous contents of organic crops [2–4]. One review [3] also reported total nitrogen levels and found higher levels in conventional crops. These findings per se are not nutritionally relevant for humans. However, phosphorus and nitrogen have key roles in many aspects of plant metabolism. One can therefore hypothesize substantial changes in various plant metabolic pathways, and an impact of the production system on the metabolome, as a consequence of differential phosphorus and nitrogen uptake by the plant. While phosphorus is a component of nucleic acids, a key regulator of enzyme activities, a bio-membrane component, and is essential for energy transfer in plants, nitrogen is an integral constituent of proteins, nucleic acids, chlorophyll, co-enzymes, and secondary metabolites and is a regulator of phytohormone activity in plants [5]. Thus, we should expect to find effects on the plant metabolome as a consequence of organic vs conventional agricultural management practices.

One drawback of targeted nutrient analysis is the fact that major differences of plant composition may remain undetected, bearing in mind that plants contain thousands of metabolites. Furthermore, there is a growing awareness that in order to adequately describe the nutritive value of a foodstuff, nutrients should not be considered separately, but as embedded in their food matrix [6, 7]. Therefore, changes in the food matrix as a function of agricultural production system may be as interesting as changes of the nutrient profile itself. Also, conclusions on the biological reasons for observed nutrient differences in plant foods, such as the up- or downregulation of specific metabolic pathways in response to the agricultural production regime, are difficult to draw if only small numbers of nutrients are measured.

Metabolomic data of organically produced plants may be valuable for several purposes. From a market perspective, differences in metabolite patterns of food from different production systems are of interest in assuring the authenticity of food products marketed as organic [8]. In a plant science perspective, key factors controlling plant chemical composition and thereby plant quality may be identified by metabolomics, enabling targeted optimization of agricultural management practices aimed at generating high-quality crops. The detection of differences in food composition as a function of agricultural production system is also of interest with respect to health research. Some animal studies have found an impact of the production system of the feed on certain animal health parameters [9]. In a longer perspective, it may be possible to use detailed information on differences in food composition as a starting point for investigations into the mechanism of differential health effects.

There are only a few studies that make use of large-scale non-targeted analysis techniques in analyzing differences of organic and conventional crops. One study compares protein profiles in potato tubers in one growing season [10]. Of the 1,100 proteins detected, 160 (15 %) were differentially expressed in potatoes grown under organic and conventional conditions, with the fertilization regime being the main source of difference. In contrast, another study comparing the proteome of organic and conventional white cabbage and carrots found only approximately 5 % of the proteins being differentially expressed as a function of farming methods [11]. Another study investigated the impact of the amount and type of nitrogen fertilization (ammonium nitrate and farmyard cattle manure) on the grain endosperm transcriptome in winter wheat [12]. This study found that “gene expression is significantly influenced by the amount and form of nitrogenous fertilizer.” Findings from two of these three studies indeed show that there are differences in the plant developmental biology as a function of production system, which are relevant in the context of agricultural practices. These differences are expected to manifest themselves also as differences in the metabolome of the crop, i.e., as measurable differences in nutrient (or non-nutrient or anti-nutrient) content, as a function of the production system.

A few studies have applied non-targeted metabolomics to organic and conventional crops [13–15]. These studies report a systematic effect of the production system on the metabolome of pepper, tomatoes, ketchup, and maize kernels based on farm pairings or market-basket samplings. Another metabolomics study [16] reports a separation of conventional and organic red grapefruit samples from a well-designed field study using a large number of samples from 2 years, using a descriptive (“ANOVA-PCA”) rather than a predictive analytical approach. Thus, previous studies have shown that the metabolome of organic and conventional crops may be different; however, based on the limited number of studies available, it remains impossible to draw general conclusions.

The objective of this study was to explore the effect of organic and conventional growing conditions on the overall chemical composition of a crop using non-targeted metabolomics. White cabbage was used as a model crop due to the high nitrogen demand of plant species from the Brassicaceae family [17]. To our knowledge, this is the first study that investigates the influence of organic vs conventional agricultural management practices on a crop’s metabolome based on a rigidly controlled long-term field trial and predictive statistics.

## Material and methods

### Samples

Cabbage samples (*Brassica oleracea* L. convar. *capitata* (L) Alef. Var. *alba* DC ‘Impala’) were obtained from the Danish field trial study “VegQure” in two consecutive years (2007 and 2008) [18]. In VegQure, a traditional conventional growth strategy (C) relying on use of synthetically produced nitrogen fertilizers and pesticides were compared to several organic strategies (O1 and O2) in an 8-year crop rotation conducted on a sandy loam on one field site (Aarslev, 10° 27′ E, 55° 18′ N). The organic systems were managed in compliance with the European Union guidelines for organic farming [19, 20]. The O1 system relied on import of animal manure while the O2 system received nutrients via green manures and catch crops as well as a small amount of animal manure to satisfy the high-nitrogen demand of cabbage. Total N/P/K fertilization rates in kilogram per hectare were for cabbage on average across 2 years: 310/45/145 (C), 225/20/85 (O1), and 135/10/50 (O2). All field plots were treated with the biological insecticide *Bacillus thuringiensis*. The C plots were treated with the synthetic insecticides pirimicarb and alpha-cypermethrin and the synthetic fungicide azoxystrobin (2008). All plants were irrigated during a dry and warm period of 2008 to avoid drought stress-related yield reductions and to reduce seasonal variations. All field plots used for the organic systems (O1 and O2) were managed organically for a decade prior to the experiment, and the conventional (C) plots were established in 2005. The three systems were conducted in triplicate, thereby yielding a total of 18 independent cabbage samples (3 systems × 3 replicate plots × 2 years). Further details regarding field trial characteristics such as soil type, soil fertility, crop rotation, sowing and harvest dates, fertilizer rates, pesticide applications, climate data, etc. can be found in [18] and in the supporting material of [21].

At maturity, cabbages from all three systems were harvested at the same day. One sample consisting of four cabbage heads was taken per plot. In addition, duplicate sampling was conducted for every third plot in order to test the representativeness of the sampling strategy (“sampling duplicates”). All cabbage heads were stored at 1 °C and 100 % humidity until sample preparation (<6 weeks). All samples from each year were prepared on the same day. Each cabbage head was washed in milli-Q-water, cut into eight pieces with a ceramic knife, and half of the pieces were pooled with four pieces from three other cabbages, thereby representing one sample. The whole sample was cut into 0.5-cm wide slices, washed in milli-Q water, and was frozen at −20 °C followed by freeze-drying for 48 h. Afterwards, the samples were crushed and homogenized in a plastic bag and stored at −20 °C in an inert nitrogen atmosphere until sample extraction in spring 2010 (1.5 or 2.5 years). Sample preparation was conducted in a fully randomized way in all steps from harvest to homogenization.

### Elemental analysis

The content of P, K, Mg, and S was measured by inductively coupled plasma–optical emission spectroscopy (ICP-OES, Optima 5300DV, PerkinElmer, MA, USA) and the content of C and N using IRMS (ANCA-SL elemental analyzer coupled to a 20–20 Tracermass mass spectrometer, Sercon Ltd., Crewe, UK.) as previously described in detail by Laursen et al. [22].

### Reagents and chemicals

Extraction solvents: water was supplied by a Millipore (Solna, Sweden) system. Methanol (Chromasolv for gradient elution) and acetone (Chromasolv for HPLC) were purchased from Sigma-Aldrich (Stockholm, Sweden).

Chromatography solvents: Water was supplied by a Millipore system. Acetonitrile (ACN) (LC-MS Chromasolv grade), ammonium formate (for LC-MS), and formic acid (for LC-MS) were obtained from Sigma-Aldrich (Stockholm, Sweden).

### Sample extraction

Samples were extracted and analyzed in randomized order in duplicate (technical replicates). Approximately 100 mg of freeze-dried cabbage were transferred into 15-mL centrifugal tubes made of polypropylene (Sarstedt, Helsingborg, Sweden) in a glove bag containing a dry nitrogen atmosphere. Samples were extracted in two batches. Six replicate extraction quality control (extraction QC) samples were included per batch, one extraction QC after every fourth sample, in order to account for batch-to-batch variation in extraction efficiency and to measure overall method precision (see Electronic Supplementary Material Table S2). These extraction QC samples were pooled cabbage samples. After every 10 samples, one extraction blank was included.

Samples were sequentially extracted four times. Extraction solvents for the four sequential extractions were the following: first and second extractions, 1 mL MeOH/H2O 50/50 *v*/*v*; third and fourth extractions, 1 mL acetone/H2O 70/30 *v*/*v*. After solvent addition, samples were vortexed. Samples were then placed in an ultrasonic bath at 20 °C for 30 min. Samples were centrifuged at 4,000×*g* for 5 min at 20 °C in a Sigma 4K15 centrifuge (Sigma Laborzentrifugen, Osterode am Harz, Germany). Supernatants were decanted; sequential extracts were pooled for each sample and stored during the remaining extraction process on a water/ice bath at 0 °C. Extracts were filtered through 0.45-μm syringe filters (Millex-LCR, PTFE, 13 mm, non-sterile, Millipore, Solna, Sweden). Filtered extracts were stored in aliquots at −80 °C until analysis.

### Sample analysis

Prior to analysis, 300 μL of sample extract were evaporated in a Speedvac evaporator and reconstituted in 60 μL MeOH/H_2_O 50/50 *v*/*v* containing 5-mM ammonium formate buffer, pH 3.75. One microliter of reconstituted extracts were analyzed by LC-MS on an Agilent Infinity 1290 UHPLC system coupled to an Agilent 6540 Q-TOF mass spectrometer (Agilent Technologies, Kista, Sweden).

Samples were handled by an Agilent 1290 autosampler, and separation was achieved using an Ascentis Express C18 precolumn and column kept at 40 °C (length 15 cm, diameter 2.1 mm, particle size 2.7 μm, pore size 90 Å, Sigma-Aldrich, Stockholm, Sweden). The LC system was coupled to the mass spectrometer via a “Jet Stream” electrospray interface. Mobile phase A was water/ACN 95/5 % *v*/*v* with 5-mM ammonium formate buffer pH 3.75. Mobile phase B was water/ACN 5/95 % *v*/*v* with 5-mM ammonium formate buffer pH 3.75. The mobile phase flow rate was 0.4 mL/min and the gradient was 0–1 min 0 % B, 1–10 min 0–100 % B, 10–15 min 100 % B. Injection needle washing was done by dipping the needle consecutively into three 2-mL glass vials, containing 100 % B, 100 % A, and again 100 % A. Samples were separately analyzed in both positive and negative modes. Electrospray ionization voltages were +4 kV (positive mode) and −4 kV (negative mode) with nozzle voltages of +1 and −1 kV, respectively. Nitrogen was introduced as a sheath gas at 9 L/min at 350 °C and as countercurrent dry gas at 8 L/min and 300 °C. The rough vacuum was approximately 3 mbar and the quadrupole vacuum was approximately 5 × 10^−5^ mbar. The fragmentor was set to ±100 V. The acquisition range was 50–1,700 *m*/*z* with a scan rate of 6 Hz and a sampling rate of 4 GHz. The mass accuracy was <1 ppm, and the mass resolution was >20,000 according to the tuning that was performed before the experiments. Reference ions were used for *on the fly* mass correction. LC-MS data were recorded as profile and centroid data in Agilent .d format using Agilent MassHunter Workstation version B.04.00 acquisition software (Agilent Technologies, Kista, Sweden). Every 10th sample injection was done with an injection QC sample, which consisted of a pool of extracts from over 20 samples. This injection QC was used for monitoring the overall performance of the LC-MS system and for establishing the precision of the analytical method, but not for correction of intensities.

### Data pre-treatment

Data files were converted to mzXML format using the software tool Trapper (Aebersold Lab, Seattle Proteome Center, USA) version 4.3.1, extracting only centroid data. Peak integration and peak alignment were performed using the software XCMS [23] version 1.30.3 run in R [24] version 2.14.1. Peaks were detected and integrated using centWave algorithm (parameters: ppm = 10, snthr = 5, peakwidth = c(5.15), mzdiff = −0.01, prefilter = c(6,100), fitgauss = TRUE) and aligned using Obiwarp (parameters: distFunc = "cor", profStep = 1, grouping parameters: bw = 1, mzwid = 0.005, minfrac = 0.5). In a few cases, e.g., for some molecular features with high fold change and high significance, integration and alignment were manually confirmed and were in good agreement with XCMS results. The peak lists for positive and negative electrospray data contained 7,027 and 3,096 peaks for each sample, respectively. These peak lists were cleaned from some noise: the mass spectrometer produces a “tail” of peaks for high-intensity ions (also known as “ringing”), giving rise to up to seven observed additional signals on the higher *m*/*z* side of the actual peak at typical distances of +0.01 to +0.2 amu from the actual peak and at typical intensities of 10 % or less of the actual peak. Therefore, all peaks having a retention time within 0.5 s and a *m*/*z* shift below +0.3 amu and less than 20 % intensity as another peak were removed. Also, peaks with a ratio of median intensity of all samples and median intensity of extraction blanks less than five were removed as blank signals. Furthermore, peaks with a retention time less than 1 min were removed (eluted from the column in the void volume). These cleaning steps decreased the positive and negative peak lists to 3,981 and 1,910 peaks, respectively, resulting in one combined peak list containing 5,891 molecular features for each of 48 samples. This corresponds to approximately 1,600 compounds, as estimated using the Molecular Feature Extractor of the MassHunter software. Data for one positive mode sample and one negative mode sample were missing.

In order to account for sample-to-sample variation of the sensitivity of the mass spectrometer, peak intensities within samples were median-centered to a common value (sample-wise normalization). In order to account for batch-to-batch variation in the sample extraction, peak intensities of samples from extraction batch 2 were scaled using extraction QC samples. The ratio of median values of batch 2/batch 1 of those extraction QC samples was used as a scaling factor for each variable (variable-wise correction). Finally, all technical and sampling replicates were averaged, resulting in data for 18 samples and 5,891 molecular features for the data analyses.

### Univariate data analysis

The influence of the agricultural production system on the concentration of individual compounds was investigated using two-way ANOVA with the production system and year as factors. Fold changes (ratios of relative concentrations) were reported as averaged over years. Due to the high number of tested hypotheses (number of molecular features, 5,891), the multiple testing problem was addressed. Probabilities were summarily presented as unadjusted *p* values and as adjusted Benjamini–Hochberg false discovery rates (FDR), using an adaptive variant that takes into account the estimated number of true null hypotheses by means of quantile plots [25]. Univariate data analysis was performed using the software R [24] version 2.14.1.

### Identification of compounds

The databases Metlin [26, 27], KnaPSacK [28], MassBank [29], KEGG [30], and PlantCyc [31] were used for matching of observed molecular features by molecular mass or formula. Chemical formulas were generated, and identification was attempted by making use of exact mass (<1 ppm), isotopic patterns, the Molecular Feature Extractor and the formula calculator in the MassHunter software, and intensity correlation coefficients over samples in order to identify fragments and adducts.

### Multivariate data analysis

All variables (molecular features) were scaled to unit variance. Principal component analysis (PCA) [32, 33] was initially used for unsupervised multivariate analysis. Subsequently, Orthogonal Projection to Latent Structures–Discriminant Analysis (OPLS-DA) [34] was used for supervised multivariate analysis. PCA and OPLS analysis were performed using the software Simca-P+ version 12.0.1 (Umetrics, Umeå, Sweden).

In OPLS, a sevenfold internal cross-validation (CV) was used, where 1/7th of the samples were predicted using a model based on the remaining 6/7th of the samples. This was repeated seven times so that all samples were predicted once using a model not based on themselves. All OPLS-DA models are presented with the number of components suggested by the Simca-P+ software based on the predictive performance from the internal cross-validation of the model unless specified differently. Statistical significance was assumed at *p* < 0.05.

Receiver-operating characteristic (ROC) [35] curves were used to visualize the tradeoff between sensitivity (the fraction of true positives) and specificity (fraction of true negatives) for different cutoff values for discriminating between two groups. The area under the ROC curve (AUC) defines the overall ability of the test. A useless test (not better than random classification) has an AUC of 0.5. A perfect test has an AUC of 1.00. ROC curves were obtained using the R [24] package “verification” [36].

## Results and discussion

### Field observations, sample characterization, and elemental analysis

The production of white cabbage samples was managed according to the best practice within organic or conventional plant production and, in the case of the O1 and O2 samples, in full compliance with the European Union guidelines for organic farming [20, 19]. This was reflected in the harvest yields which represented normal yields according to Danish standards. The yields from the conventional system (C) were on average, across 2007 and 2008, 16 % higher than from the animal manure-based O1 system (Table 1). However, the harvest yields from the O2 system, which was based on moderate amounts of animal and green manures, were not significantly different from the two other systems. The cabbage heads from the conventional system were visually bigger throughout the whole growing season. This observation was confirmed by the unit weight determination after harvest, which showed that conventionally produced cabbages were significantly bigger. A significant effect of harvest year was observed across systems for the unit weight which was also reflected in the harvest yields. This is suggested to be due to the weather differences between 2007 and 2008. The year effect observed for harvest yield and unit weight was negatively correlated with the concentration of N, P, K, and Mg. This indicates a dilution of these due to the high biomass production in 2008; however, all nutrient concentrations except N were within the normal range [17]. The N concentrations from 2008 indicated that plants were marginally N deficient. No systematic and significant differences were found between systems for the percentage of dry matter or the concentration of N, P, K, S, and Mg. This is in correspondence with previous studies comparing the elemental composition of organic and conventional crops [22]. Thus, the initial characterization of the produced cabbages indicated that the elemental composition was primarily affected by harvest year and not the agricultural production system. Thus, other analytical methods were required to discriminate between organic and conventional cabbages.

**Table 1:** Harvest yield, unit weight, percentage of dry matter, and plant nutrient concentrations presented as average ± standard deviation (*n* = 3 plots per system and year). *p*(system) and *p*(year) are *p* values of the two-way ANOVA with system (C, O1, O2) and year (2007 and 2008) as factors (*n* = 6 per system or *n* = 9 per year). Where *p*(system) <0.05, systems not sharing a common superscript row-wise are significantly different (Student’s *t* test, *n* = 3 per system) for that year

	Year	System			*p*(system)	*p*(year)
		C	O1	O2		
Yield (1,000 kg FW/ha)	2007	87.2 ± 7.6^a^	67.6 ± 8.3^b^	72.5 ± 9.3^ab^	0.0023	1.3 × 10^−5^
	2008	105.3 ± 1.2^a^	92.0 ± 2.5^b^	93.5 ± 9.7^ab^		
Unit weight (g FW)	2007	3213 ± 325^a^	2755 ± 147^b^	2783 ± 225^b^	0.00077	5.2 × 10^−7^
	2008	3969 ± 104^a^	3614 ± 28^b^	3409 ± 152^b^		
DM (%)	2007	9.61 ± 0.38	9.42 ± 0.26	9.79 ± 0.14	NS	NS
	2008	9.56 ± 0.17	9.76 ± 0.39	9.73 ± 0.24		
N (%)	2007	1.97 ± 0.06	1.97 ± 0.03	2.04 ± 0.07	NS	3.8 × 10^−7^
	2008	1.76 ± 0.06	1.65 ± 0.11	1.62 ± 0.01		
P (%)	2007	0.29 ± 0.02	0.28 ± 0.01	0.29 ± 0.02	NS	0.0031
	2008	0.27 ± 0.01	0.26 ± 0.01	0.25 ± 0.03		
K (%)	2007	2.60 ± 0.12	2.49 ± 0.19	2.62 ± 0.17	NS	0.0072
	2008	2.35 ± 0.08	2.26 ± 0.11	2.05 ± 0.49		
S (%)	2007	0.63 ± 0.02	0.63 ± 0.03	0.60 ± 0.03	NS	NS
	2008	0.62 ± 0.03	0.60 ± 0.03	0.56 ± 0.04		
Mg (%)	2007	0.12 ± 0.00	0.12 ± 0.01	0.12 ± 0.01	NS	0.00014
	2008	0.11 ± 0.00	0.10 ± 0.01	0.10 ± 0.00		

Nutrient concentrations are presented as weight−% (g/100 g DM). Some of these data have partially been presented earlier: Yield data have been published in the supporting material of [21] as averaged over two instead of three field plots and in [18] as averages over 3 years. DM (%), N (%), and S (%) have been presented in [18] as averages over 3 years

*NS* not significant, *FW* fresh weight, *DM* dry matter

### The metabolome of cabbage is dominated by growth year

The main objective of this work was to investigate the effect of the agricultural production system on the metabolome of the crop. Below, univariate and multivariate statistical analyses of the production system on 5,891 molecular features detected in the white cabbage sample are presented.

#### Univariate analysis

As a first step, the effect of the production system and the production year on each of the 5,891 variables (molecular features) was investigated by two-way ANOVA. This analysis is summarized in Table 2. We found that C and O2 were the systems with the largest differences. O1 and O2 samples appeared indistinguishable, with this low number of samples. Statistical power was therefore gained by pooling O1 and O2 samples due to increased sample size. Of the 5,891 molecular features, 110 were present in different concentrations in C compared to O1/O2 samples with FDR <0.05. Fold changes between C and O1/O2 systems among these 110 compounds ranged from 1.14 to 2.53 (median, 1.54). For a comparison of the magnitude of the effect of production system and production year, fold changes between 2007 and 2008 year samples among the 110 most significant compounds out of the 2,359 compounds with FDR <0.05 ranged from 1.18 to 6.45 (median, 2.05). In Electronic Supplementary Material Fig. S1, we present Venn diagrams showing that the overlap of molecular features that are influenced by the production system and by the production year (for FDR <0.05 and FDR <0.10) is close to what would be expected by chance.

**Table 2:** Summary of two-way ANOVA with production system and year as factors based on 5,891 variables (molecular features). Presented is the number of significantly different variables using various criteria for statistical significance in various comparisons of systems

	C vs O1/O2	C vs O1	C vs O2	O1 vs O2	C vs O1 vs O2	2007 vs 2008
*n* (number of samples)	6 + 12	6 + 6	6 + 6	6 + 6	6 + 6 + 6	9 + 9
ANOVA *p* < 0.001	74	3	27	1	21	778
ANOVA *p* < 0.05	1,223	470	1,380	240	820	2,438
FDR <0.05	110	0	0	0	0	2,359
FDR <0.10	605	0	1,173	0	0	3,070
Estimated number of false null hypotheses (true differences)	2,489	1,741	2,951	0	2,449	3,346

#### Compound identification

The 110 molecular features with FDR <0.05 in the comparison of C vs O1/O2 samples from Table 2 originated from 46 compounds. In Electronic Supplementary Material Table S1, we present observed ions, proposed formulae and identities, relative concentrations in conventional vs organic samples and level of identification of the 20 compounds with highest statistical significance. Analytical precision data for these compounds are presented in Electronic Supplementary Material Table S2. The median overall precision (extraction and LC-MS analysis) of the 20 compounds with highest statistical significance was 12.7 %. The median overall precision of all 5,891 molecular features was 18.3 % RSD. To our knowledge, no authoritative guidelines or reference values are available for the analytical precision of compounds in untargeted metabolomics. For comparison, one metabolomics study based on solvent extraction and direct infusion–Fourier transform ion cyclotron resonance-MS reported a comparable median overall RSD of 18.0 % for 2,488 molecular features at a comparable stage of data processing (“X^TIC+batch^” in Electronic Supplementary Material Table S1 of [37]) [37].

For 13 of the 20 compounds with highest statistical significance, we suggest a molecular formula; for six compounds, we annotate a sub-structure, and for one compound, we present a putative identity. The absence of comprehensive mass spectral databases for plant metabolites complicated compound identification. Notably, sulfur-rich compounds, which are prominently present on our list and whose molecular formulae can be established with relatively high confidence due to the distinct isotopic pattern of sulfur, appear to have weak coverage in the searched databases. Of the 13 compounds with a proposed molecular formula, only two had matches in the databases.

Tandem mass spectrometry could lead to further insights into substructures, but in the absence of database coverage, it cannot lead to identification. The next step in the identification process would therefore be the chemical isolation and de novo structure elucidation. Although several combinations of tools are available for this purpose, elucidation of unknown structures remains a challenging task in plant metabolomics [38]. As the main objective of this work was the investigation of metabolome-wide differences rather than the isolation of individual biomarkers, we did not focus on providing further compound identification at this point. Instead, we provide information about the level of identification that actually was reached in accordance with proposed guidelines of the Metabolomics Standard Initiative (MSI) [39]. However, in future studies, the identification of a number of metabolites might increase the understanding of which metabolic pathways are up- or downregulated as a consequence of the production system. Also, in a longer perspective, an improved mechanistic understanding of how biomarkers with potential positive health effects are influenced by different cultivation systems could be gained.

#### Principal component analysis discriminates growth years

**Fig. 1:**
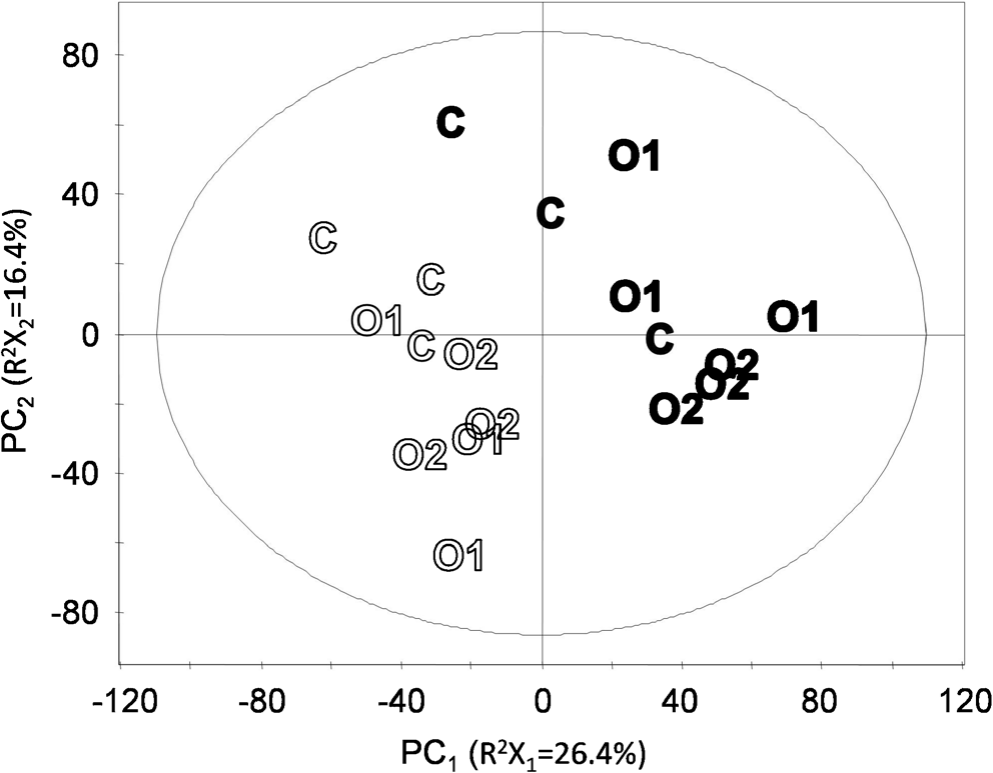
Scores plot of PCA of all 18 samples and 5,891 variables. Displayed are scores of PC_1_ and PC_2_. Production year, *open symbols*: 2007; *filled symbols*: 2008. Ellipse: Hotelling’s T^2^ (0.95)

A principal component analysis (PCA) of metabolomics data of all samples revealed that the first and second principal components (PCs) clearly separated samples by production year (Fig. 1). The same principal components also largely separated samples of production systems C and O2, with O1 samples overlapping with the other systems (Fig. 1). The first two PCs described 42.8 % of the variation between samples. Classification boundaries in this plot between samples from different years and between samples from C and O2 samples were approximately orthogonal. Accordingly, there were independent effects of the production year and of the production system on the cabbage metabolome.

#### Classification analysis

PCA allows for an analysis of which factors are responsible for the largest part of the variation of the data. In contrast, supervised models can be used to understand whether samples can be separated using a specific factor. Therefore, based on the same dataset, supervised OPLS-DA models were built to distinguish between production systems (Table 3).

**Table 3:** Summary of OPLS-DA models for the distinction of samples from different classes

Model number	Class 1	Class 2	Class 3	Number of components	*R* ^2^ *Y*(cum)	Q^2^(cum)	*p*(Cross-validation-ANOVA)	Correct classification rate in internal cross-validation
Models based on 5,891 variables (full dataset)								
1	C	O1	–	1 + 7 + 0	1	0.499	0.99	12/12 = 100 %
2	C	O2	–	1 + 1 + 0	0.887	0.500	0.24	10/12 = 83 %
3	O1	O2	–	0 + 0 + 0	–	–	–	–
4	C	O1/ O2	–	1 + 0 + 0	0.595	0.307	0.064	14/18 = 78 %
5	C/O1	O2	–	1 + 0 + 0	0.528	0.0636	0.61	10/18 = 56 %
6	C/O2	O1	–	0 + 0 + 0	–	–	–	–
7	C	O1	O2	1 + 1 + 0	0.306	0.140	0.34	–
8	2007	2008	–	1 + 1 + 0	0.960	0.856	2.2 × 10^−5^	18/18 = 100 %
Models based on 2,796 variables (refined dataset)								
9	C	O1	–	1 + 1 + 0	0.950	0.333	0.52	10/12 = 83 %
10	C	O2	–	1 + 0 + 0	0.781	0.589	0.018	11/12 = 92 %
11	O1	O2	–	0 + 0 + 0	–	–	–	–
12	C	O1/ O2	–	1 + 0 + 0	0.668	0.442	0.013	15/18 = 83 %
13	C/O1	O2	–	1 + 0 + 0	0.565	0.207	0.18	13/18 = 72 %
14	C/O2	O1	–	0 + 0 + 0	–	–	–	–
15	C	O1	O2	1 + 0 + 0	0.340	0.220	0.12	–
16	2007	2008	–	0 + 0 + 0	–	–	–	–
External validation based on 2,796 variables (refined dataset)								
			Validation samples				Correct classification rate in external validation	
17	C (2007)	O1/O2 (2007)	2008	1 + 0 + 0	0.724	0.293	7/9 = 78 %	
18	C (2008)	O1/O2 (2008)	2007	1 + 5 + 0	1.00	0.825	8/9 = 89 %	
Mean of 17 and 18	C	O1/O2	–	–	–	–	83 %	

The slash “/” symbol indicates that samples from several classes that have been pooled; model performance: *R* ^2^ *Y*(cumulative) (perfect model: *R* ^2^ *Y*(cum) = 1) is a measure of the descriptive performance of the model; *Q* ^2^(cumulative) (perfect model: *Q* ^2^(cum) = 1), *p*(cross validation-ANOVA) (perfect model: *p* = 0), and the correct classification rate (perfect model: 100 %) are measures of the predictive performance of the model; *p* is the probability that the model may be the result of just chance based on internal cross-validation

**Fig. 2:**
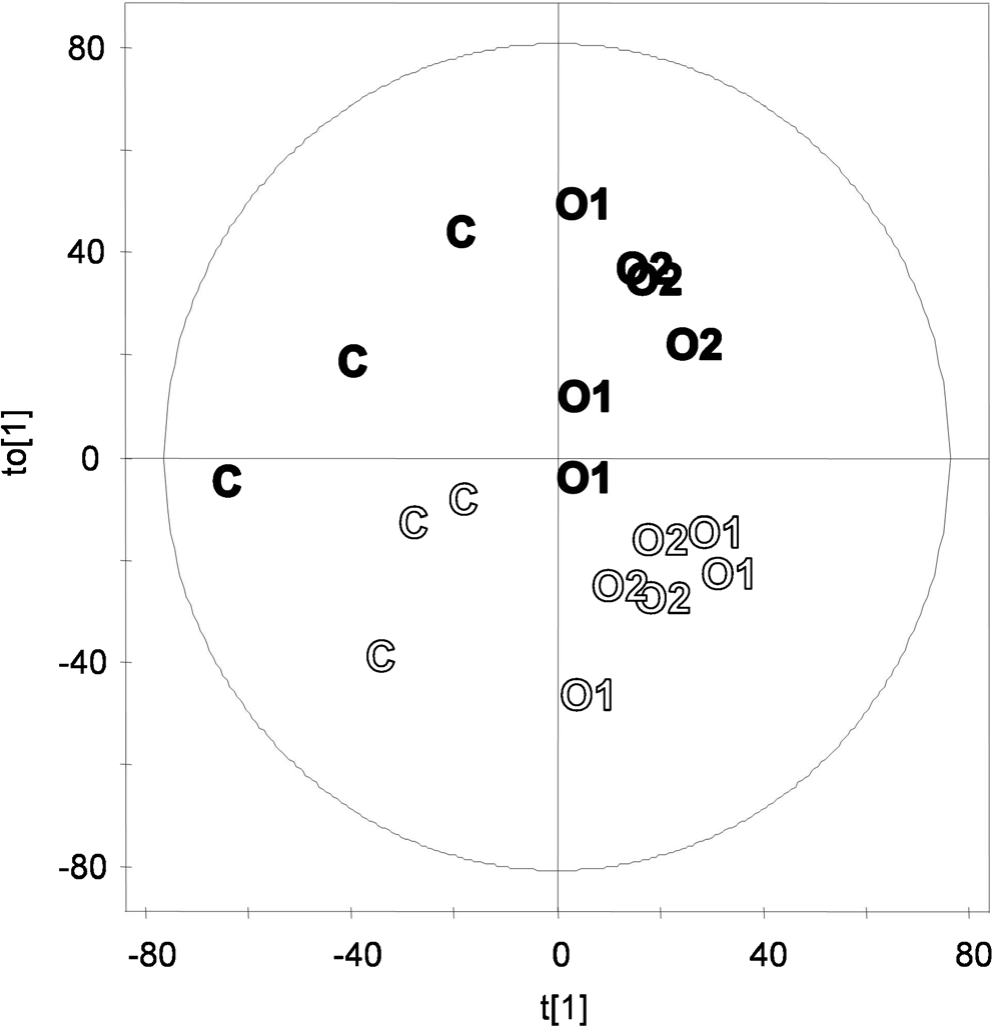
Scores plot of OPLS of model 4, discriminating C and O1/O2 samples. *n* = 18; 5,891 variables. The first predictive component (t_1_) and the first orthogonal component (t_o1_) are shown here. Production year, *open symbols*: 2007; *filled symbols*: 2008. Ellipse: Hotelling’s T^2^ (0.95). *R* ^2^ *X*_1_ = 0.143, *R* ^2^ *X*_Xside comp 1_ = 0.231

Figures of merit of the internal cross-validation are shown in Table 3. C and O2 as well as C and O1 samples could be distinguished by OPLS models (i.e., the Simca software suggested an OPLS model based on a positive Q^2^(cum) value), but not with statistical significance. O1 and O2 samples were too similar, so no OPLS model was suggested by Simca. In order to gain statistical power, we therefore pooled O1 and O2 samples and compared these samples from different organic production systems to the conventional system C. The resulting model could not significantly distinguish between organic and conventional cabbage (*p* = 0.064) (Fig. 2, Table 3 model 4). A highly significant (*p* = 2.2 × 10^−5^) OPLS model could be built for the classification of samples by production year (model 8).

Supervised classification models generally carry a risk of over-fitting and need to be carefully validated. Initially, we chose an internal cross-validation of the models. Further below, an external validation of a refined dataset is presented.

### The agricultural production system is reflected in the metabolome of cabbage

Of the 5,891 variables in the dataset, a large number likely consists of noise with respect to a classification into different production systems. It was desirable to reduce this noise in order to isolate the information that carries classification power important for the distinction between production systems and in order to provide a reliable means of sample classification. If, however, variables were removed from the dataset simply based on a non-significant contribution to a model distinguishing between production systems (here, e.g., model 4 in Table 3), a risk of over-fitting would be introduced because variables with a false-positive contribution to the model would be enriched in the remaining dataset.

**Fig. 3:**
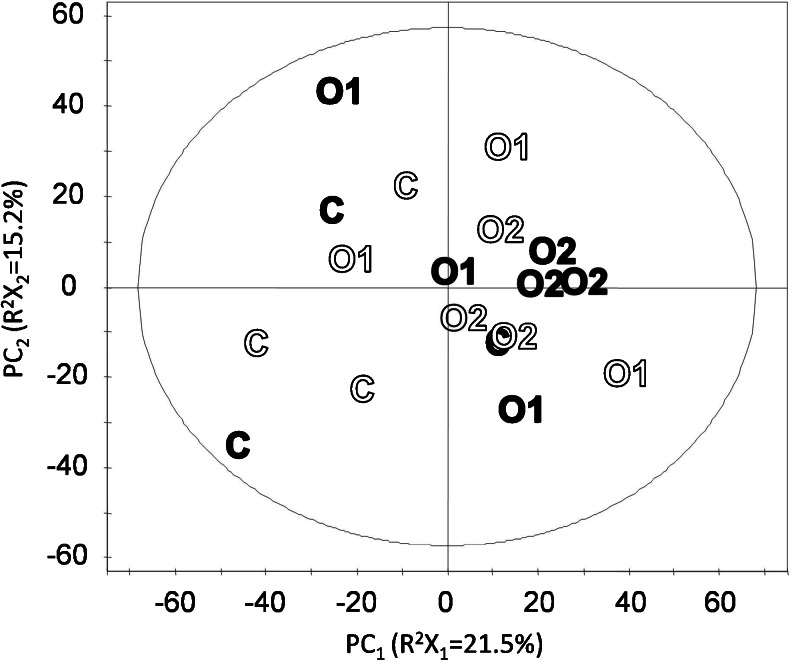
Scores plot of PCA of refined dataset: 18 samples and 2,796 variables. Displayed are scores of PC_1_ and PC_2_. Production year, *open symbols*: 2007; *filled symbols*: 2008. Ellipse: Hotelling’s T^2^ (0.95)

Instead, we chose to focus on removing the large contribution of the production year on the metabolome (Figs. 1 and 2 and Table 3, model 8), thereby avoiding the risk of enriching variables with a false-positive contribution with respect to a distinction between production systems. In Fig. 2, it is apparent that samples that are separated by production system along a predictive (horizontal axis) component also separate in an unsupervised way by production year along the first orthogonal component (vertical axis). This confirms an influence of the production year on the sample metabolome that is orthogonal to the influence of the production system, which is consistent with the observation made earlier using PCA (Fig. 1). Model 8 in Table 3 classified very successfully (*p* = 2.2 × 10^−5^) the samples from the two production years. In model 8, 3,095 variables had a significant contribution to the classification based on the variable importance in the projection (VIP). These variables were removed from the dataset, and PCA and OPLS models distinguishing samples from different production systems were built on a dataset containing the remaining 2,796 variables (Fig. 3, Table 3).

Figure 3 shows the first two components of a PCA of all samples on the reduced dataset containing 2,796 variables. PC_1_ (*R* ^2^ *X*_1_ = 0.215) largely separates samples from C and O2, with O1 samples overlapping with samples from the other two systems. After removal of variables describing the variation between years, the production system is now the most important source of variation between samples.

A comparison of results in Table 3 [models based on 5,891 variables (full dataset) and models based on 2,796 variables (refined dataset)] reveals that all *p* values and correct classification rates improved using a reduced data set with fewer variables. Hereby, the distinctions between C and O2 samples (*p* = 0.018) and between C and pooled O1/O2 samples (*p* = 0.013) became statistically significant.

After removing the variation due to the production year, we also attempted a prediction of the production system of samples from 2008 using an OPLS model built on samples from 2007 and vice versa (Table 3). This must be regarded as an extensive external validation, although it should be noted that the refinement of the dataset, i.e., the removal of variables that are important in the distinction between production years, was done using samples from both years. We chose to pool the samples from the two organic growing systems for this purpose because these samples have been indistinguishable in all previous models.

Table 3 shows results of the external validation of OPLS models classifying samples into C and O1/O2 samples. The figure of merit of this external validation is the correct classification rate. On average, 83 % of samples have been correctly classified into being of conventional or organic origin using OPLS models built on samples from one year and predicting samples from the other year. It should be noted that this validation is still based on the 2,796 variables, many of which likely constitute noise with respect to a classification of samples into production systems. A further refinement of these models by the selection of variables will probably result in a better predictive performance. However, such refinement appears only meaningful if further samples for external validation were available. If metabolomics was to be used for food authentication purposes, such a refinement, based on a much larger set of samples would be warranted.

ROC curves can be used for selecting a suitable tradeoff between sensitivity and specificity in a classification. For example, in a food authentication setting, as many conventional samples as possible should be identified among samples labeled as organic (sensitivity or true positive rate), but it is very important that samples of organic origin are actually classified as organic (specificity or true negative rate) and not falsely as conventional. One would therefore choose a specificity close to one and could read from the ROC curve the achievable sensitivity based on the underlying classification model.

The ROC curves of the external validation, based on predicted scores, are shown in Electronic Supplementary Material Fig. S2. The ROC curve for model 17 had an AUC of 0.889 (*p* = 0.048), and the ROC curve of model 18 had an AUC of 1 (*p* = 0.012). These ROC curves could be regarded as indicative of the potential performance of classification models based on metabolomics and OPLS for food authentication purposes provided that other factors (geographical, crop cultivar, etc.) could successfully be included in such models.

In summary, we were able to distinguish between samples from conventional and organic production using samples from a rigidly controlled field trial and a multivariate analysis approach aimed at avoiding over-fitting. We can conclude that the production system has a measureable impact on the metabolome of the samples from this specific study. This is confirmed by a successful external validation, which is a demanding way of validating classifiers.

### Multiple comparisons

A well-known statistical problem is the treatment of multiple comparisons. If many hypotheses are tested, inevitably some null hypotheses will be rejected just by chance (e.g., 5 % of null hypotheses if the chosen level of significance for individual hypotheses is *p* < 0.05). The most appropriate way of treating this problem may be different in different contexts. If the emphasis is on the estimation of effects (e.g., in a systematic review with several reported clinical outcomes of a treatment), adjustments for multiple testing may not be warranted [40] due to the associated loss of statistical power. If the focus is on a rigorous test of individual hypotheses (e.g., in the discovery of individual biomarkers), conservative family-wise error correction methods are warranted, such as the Bonferroni correction. In between, the control of the false discovery rate (FDR) is a less conservative adjustment. An alternative is the definition of one or very few primary hypotheses.

For the univariate part of the analysis, we chose to summarily present *p* values for pair-wise comparisons of classes as unadjusted and using FDR (Table 2). A more conservative approach appears unnecessary here because our aim was not the discovery of individual biomarkers for organic or conventional production. We did not adjust for multiple comparisons of sample classes (six such comparisons in Table 2): we consider the primary comparison to be C vs O1/O2 samples, i.e., samples from organic vs conventional agriculture. Accordingly, models 4 and 12 (refined) are the primary comparisons in the multivariate part, and *p* values in Table 3 are not adjusted.

So far, the multiple testing problem has only rarely been addressed in the context of the composition of foods from organic and conventional agriculture. Notably, recent reviews come to different conclusions whether many [1] or few [2] significant (micro)nutrient differences are detected between organic and conventional crops, apparently due to the application [2] or non-application [1] of family-wise error correction in the meta-analysis of several reported outcomes (nutrient concentrations). In any case, even if statistically significant, differences in nutrient levels were rather small and variation between studies was large [1].

### The biological interpretation of the observed effects

Ideally, samples from the various production systems should be harvested at the same physiological age (ripeness). In case the physiological age is influenced by the production system, harvesting at the same time, rather than at the same physiological age, would yield results where the effects of production system and degree of ripeness on the crop’s composition are confounded. In the present case of autumn-harvested white cabbage, in the late autumn, physiological age is primarily affected by acclimation to the coming winter conditions due to low temperature and short day lengths. Accordingly, this study’s cabbage samples likely represent a valid comparison of the effect of the production system that is not confounded by a different degree of ripeness.

The main rationale for observed differences in the proteome and transcriptome studies lies in the availability of plant nutrients. In conventional (intensive and high input) agriculture, mineral fertilizers, often in combination with animal manures, are the predominant source of plant nutrients. Most mineral fertilizers are soluble in the soil solution and readily available to plants. In organic agriculture, mineral fertilizers are prohibited. Instead, essential plant nutrients such as nitrogen, phosphorous, and potassium are supplied via green and/or animal manures, which are often composted prior to application. Most plant nutrients are here initially, to a large extent, bound to organic matter and are not immediately available for plants until liberated during mineralization. Furthermore, by practice and regulations, the amount of annually supplied plant nutrients per hectare is generally considerably higher in conventional agriculture (see “Material and Methods”). Also, a different depth distribution can be expected. Systems employing green manures, like the O2 system, tend to retain plant available N closer to the soil surface than other systems.

Conventional and organic agriculture can therefore potentially be distinguished by differences in the source, amount and biological availability of essential plant nutrients, and resultant changes in the chemical composition of crops.

Under conditions of ample plant nutrient availability, the primary plant metabolism, responsible for growth processes, is generally emphasized. Under conditions of limited nutrient availability, metabolic processes of the secondary plant metabolism are upregulated. These are responsible for stress tolerance and for the diversification of plant functions such as biotic defense and ripening. Accordingly, in an agricultural context, due to lower plant nutrient provision and availability in organic compared to conventional agriculture, shifts in the balance between primary and secondary metabolism towards a stronger emphasis on secondary metabolism in organic crops are expected. On the other hand, plant producers in both organic and conventional systems strive for optimum plant growth conditions, and it could be argued that within the actual range of plant nutrient abundance and availability, plants are homeostatic, i.e., plants are able to maintain an identical metabolic state of optimum growth.

It is now being recognized that plants may alter their leaf metabolome in a direct response to an altered soil microbiome [41]. The use of pesticides in conventional agriculture is likely to impact the community of soil microorganisms [42]. Hypothetically, this represents another way of how the agricultural production system may influence the crop’s metabolome.

Based on our results, we conclude that plant homeostasis is not strong enough to keep the plant metabolome constant within the range of the production factors studied. However, changes of concentrations of single compounds between production systems were generally modest, and we did not observe drastic effects, like entire pathways switched off in certain production systems.

The metabolome differences between C and O1/O2 cabbage and the inability to distinguish between O1 and O2 samples are paralleled by the markedly higher crop yields of C compared to O1 samples, while O1 and O2 had a similar crop yield.

In contrast to many other studies and to the conclusions of reviews in this area of research [2–4], we did not observe differences in nitrogen and phosphorus concentrations of the crop as a function of production system. Yet, we did observe changes in the metabolome between organic and conventional crops across production years.

Overall, the influence of the production year on the metabolome was greater than the influence of the production system. This is apparent from the PCA of Fig. 1 and from the large number of significant differences in Table 2. Also, fold changes were larger in the comparison of years than in the comparison of production systems. However, the estimated number of differences in the comparison of production systems is almost as high in the comparison of production years. Also, the overlap of molecular features that are influenced by year and by production system is close to what would be expected by chance (see Electronic Supplementary Material Fig. S1). We interpret this as year and production system having substantial independent influences on the metabolome as a whole, influencing a similar number of metabolites, but the magnitude of the influence (as fold change) of the production year is generally larger than the influence of the production system within our experimental domain.

Despite the observed differences, we cannot draw any conclusions on which production system yields the healthier crop. Some animal studies have previously observed differential health effects of organic vs conventional feed [9, 43]. We do find differences in the crop’s metabolomics composition that may grant plausibility to observed health effects in other studies. Further studies specifically aimed at deciphering possible health effects of organic plant production are required to clarify this.

Metabolomics has also been proposed as one of several approaches for organic food authentication [8, 14]. In that context, it should be emphasized that apart from the production year (as in the present work), several other factors such as crop cultivar [44], geographical growing location [44, 45] comprising climatic conditions and soil mineralogy, post-harvest handling [46], and the range of farming methods within production systems, among others, may influence the crop’s metabolome and subsequently blur the ability to classify samples according to the agricultural production system.

A combination of metabolomics-derived biomarkers and data from other analytical techniques, such as elemental fingerprint analysis [22] or stable isotope analysis [47], could probably enhance the reliability of organic food authentications.

## Conclusions

We conclude that the chemical composition at the metabolome level of white cabbage grown in a controlled long-term field trial is influenced by organic vs conventional farming practices in a manner that is retained between production years. This adds to the growing number of reports on effects of organic vs conventional production systems on the chemical composition of crops. By measuring approximately 1,600 compounds by untargeted metabolomics, we found a systematic influence of the production system on the crop’s chemical composition. This suggests that metabolomics (or metabolomics-derived biomarkers) could potentially be suitable for authenticating the agricultural origin of organic products, possibly as a complement to other analytical techniques.

## Electronic supplementary material

Below is the link to the electronic supplementary material.

ESM 1 (PDF 735 kb)
