# Supplementary material for: Discrimination of conventional and organic white cabbage from a long-term field trial study using untargeted LC-MS-based metabolomics
Source: Anal Bioanal Chem. 2014 Mar 12;406(12):2885–97. doi: 10.1007/s00216-014-7704-0 (PMC3984666; doi:10.1007/s00216-014-7704-0)
Supplement: Supplementary file 1 — (PDF 735 kb) [file 216_2014_7704_MOESM1_ESM.pdf]

Analytical and Bioanalytical Chemistry

Electronic Supplementary Material

Discrimination of conventional and organic white cabbage from a long-term field trial study using untargeted LC-MS-based metabolomics

Axel Mie, Kristian Holst Laursen, Magnus Åberg, Jenny Forshed, Anna Lindahl,
Kristian Thorup-Kristensen, Marie Olsson, Pia Knuthsen, Erik Huusfeldt Larsen, Søren Husted

Venn diagrams

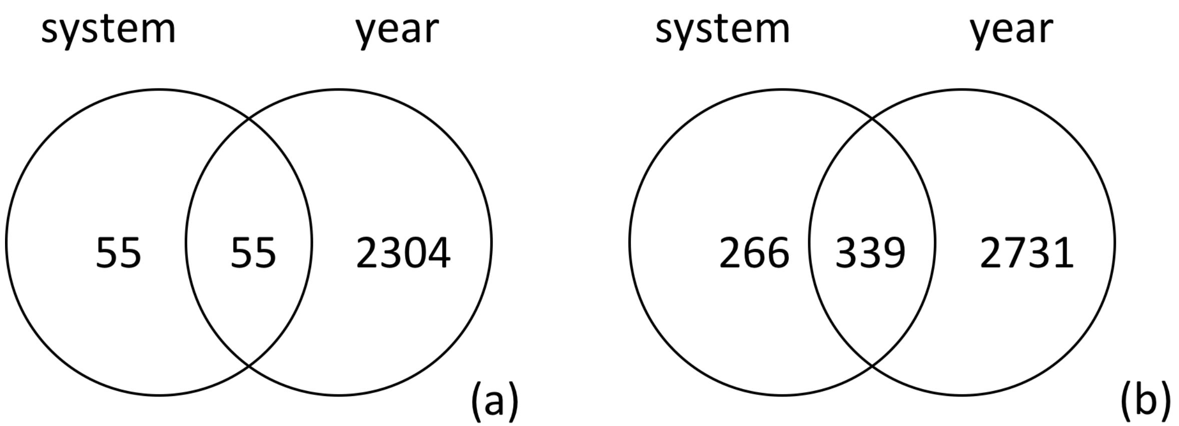

Fig. S1. Overlap of molecular features. Venn diagram of molecular features (a) significant with FDR<0,05 in the comparisons C1 vs. O1/O2 (110) and 2007 vs. 2008 (2359) (see table 2). By chance, 44 would be overlapping. (b) significant with FDR<0,10 in the comparisons C1 vs. O1/O2 (605) and 2007 vs. 2008 (3070) (see table 2). By chance, 315 would be overlapping

Table S1. 20 compounds with highest statistical significance in the comparison of production systems, based on the list of 110 molecular features differing between C and O1/O2 systems with FDR<0.05

compound number	observed m/z of molecular ion	polarity molecular ion	ion	retention time (min)	observed m/z of fragments and adduct ions (intensity % relative to molecular ion, proposed ion formula)	p-value (unadjusted)[1]	relative abundance conventional/organic[2]	proposed formula (neutral)[3]	mass error of molecular ion (ppm)	proposed identity or compound class[4]	level of identification[5]
1	581.2376	pos	+H	1.03	128.0706 (955, $[C_6H_9NO_2+H]^+$), 250.1110 (4130, $[C_{10}H_{19}NO_4S+H]^+$)	1.30E-04	1.26	$C_{24}H_{40}N_2O_{12}S$	-0.19	unknown	4
2	263.1426	pos	+H	1.14		2.67E-04	1.18	$C_{11}H_{22}N_2O_3S$	-0.84	unknown	4
3	315.0723	neg		1.16		3.83E-04	0.73	ambiguous		unknown	4
4	661.2425	pos	+H	1.65	358.0901 (1368, $[C_{15}H_{15}N_7S_2+H]^+$), 342.1162 (570, $[C_{12}H_{27}N_3S_4+H]^+$)	1.84E-04	1.39	ambiguous		unknown	4
5	293.0990	pos	+H	3.19	315.0811 (7, $[M+Na]^+$)	5.25E-05	1.19	$C_{11}H_{20}N_3O_2S_3$	-0.7	unknown	4
6	822.3179	pos	+H	4.02	844.2997 (144, $[M+Na]^+$), 660.2651 (93, $[M-[C_6H_{12}O_5+H]^+$)	2.33E-05	1.28	ambiguous		contains hexose moiety	3
7	961.3810	pos		4.40		1.82E-04	1.47	ambiguous		unknown	4
8	777.2214	pos	+Na	4.49	793.1953 (72, $[M+K]^+$), negative mode: 1507.4548 (9, $[2M-H]^-$)	4.65E-04	1.43	$C_{34}H_{42}O_{19}$	-0.19	a flavonoid glycoside	3
9	243.9954	pos	+H	4.77	179.9970 (51, $[M-CH_3SOH+H]^+$), 195.9919 (24, $[M-CH_3SH+H]^+$), 164.0198 (20, $[M-CH_4S_2+H]^+$, $[C_5H_9NOS_2+H]^+$, iberin)	2.20E-04	1.49	$C_6H_{13}NOS_4$	-0.72	an iberin conjugate	3
10	275.9675	pos	+H	5.47	211.9691 (39, $[M-CH_3SOH+H]^+$)	5.04E-06	1.62	$C_6H_{13}NOS_5$	-0.61	unknown	4
11	327.0657	pos	+H	5.76	349.0475 (7, $[M+Na]^+$), 365.0214 (8, $[M+K]^+$), 164.0198 (12, $[C_5H_9NOS_2+H]^+$, iberin), 675.1055 (2, $[2M+Na]^+$)	2.43E-04	1.39	$C_{14}H_{18}N_2OS_3$	-0.87	an iberin conjugate	3
12	307.9395	pos		6.22		1.31E-04	1.83	ambiguous		unknown	4
13	359.0377	pos	+H	6.24	381.0195 (7, $[M+Na]^+$), 396.9934 (8, $[M+K]^+$), 739.0495 (2, $[2M+Na]^+$), 164.0198 (18, $[C_5H_9NOS_2+H]^+$, iberin), 130.0651 (124, $[C_9H_7N+H]^+$), 195.9919 (1, $[C_5H_9NOS_3+H]^+$), 229.9797 (7, $[C_5H_{11}NOS_4+H]^+$), 295.0394 (1, $[C_{13}H_{14}N_2S_3+H]^+$)	2.54E-04	1.60	$C_{14}H_{18}N_2OS_4$	-0.53	an iberin conjugate	3
14	373.0532	pos	+H	6.45	395.0350 (7, $[M+Na]^+$), 767.0808 (1, $[2M+Na]^+$), 130.0651 (96, $[C_9H_7N+H]^+$), 411.0090 (7, $[M+K]^+$), 178.0355 (19, $[C_6H_{11}NOS_2+H]^+$, sulforaphane), 210.0076 (3, $[C_6H_{11}NOS_3+H]^+$), 243.9954 (12, $[C_6H_{13}NOS_4+H]^+$)	1.72E-04	1.58	$C_{15}H_{20}N_2OS_4$	-0.33	a sulforaphane conjugate	3
15	391.0095	pos	+H	6.69		3.18E-04	1.87	$C_{14}H_{19}N_2OS_5$	0.02	unknown	4
16	387.0324	pos	+H	6.92		1.93E-04	1.75	$C_{15}H_{18}N_2O_2S_4$	-0.11	unknown	4
17	261.0527	neg	-H	7.53	162.0382 (24, $[C_9H_9NS-H]^-$), in positive mode: 130.0651 (2320, $[C_9H_7N+H]^+$)	2.57E-04	1.29	$C_{13}H_{14}N_2S_2$	-0.34	unknown	4
18	564.9494	neg		10.13		3.75E-04	2.51	ambiguous		unknown	4
19	385.8431	neg		10.85	1223.3722 (208)	2.98E-04	2.42	ambiguous		unknown	4
20	954.6157	pos	$+NH_4$	12.64	959.5709 (64, $[M+Na]^+$), 975.5445 (9, $[M+K]^+$), 613.4828 (4, $[M-C_{12}H_{20}O_{10}+H]^+$, loss of 2 galactose moieties), in negative mode: 981.5790 (51, $[M+FA-H]^-$), 935.5730 (2, $[M- H]^-$)	5.08E-04	1.32	$C_{51}H_{84}O_{15}$	0.94	1,2-Di-(9Z,12Z,15Z-octadecatrienoyl)-3-(Galactosyl-alpha-1-6-Galactosyl-beta-1)-glycerol	2

[1] p-value - based on most significant molecular feature

[2] based on most abundant ion

[3] determined using accurate m/z, MassHunter's formula calculator, and isotopic pattern. Ambiguous - too low abundance (less than 3 isotopes above noise) or too high m/z or both

[4] identities - determined using KEGG, Plantcyc, Metlin, HMDB, MassBank, KNApSAcK

[5] level of identification according to MSI: 1 - identified compounds, 2 - putatively annotated compounds, 3 - Putatively characterized compound classes, 4 - unknown compounds (Sumner et al, Metabolomics (2007) 3:211–221)

Table S2. mean peak areas, and relative standard deviations (RSD), in blanks and quality control (QC) samples. The roles of the various blanks and QCs are described in sections 2.4 and 2.5 of the article

			**Precision of LC-MS system[1]**			**Precision of extraction + LC-MS system[2]**						
							**extraction batch 1**			**extraction batch 2**		**pooled**
**compound number**	**observed m/z of molecular ion**	**polarity molecular ion**	**mean peak area blank (n=4)**	**mean peak area QC (n=8)**	**QC RSD (%)**	**mean peak area blank (n=3)**	**mean peak area QC (n=6)**	**QC RSD (%)**	**mean peak area blank (n=3)**	**mean peak area QC (n=6)**	**QC RSD (%)**	**pooled QC RSD (%) extraction batches 1 + 2**
1	581.2376	pos	0	4093	**7.7**	0	4165	17.8	0	3908	10.7	**14.9**
2	263.1426	pos	15	35586	**4.5**	21	28170	11.1	44	32377	5.0	**8.2**
3	315.0723	neg	0	1383	**18.2**	0	1733	10.1	0	1744	13.9	**12.2**
4	661.2425	pos	0	3585	**9.3**	0	2311	18.8	0	2627	18.4	**18.6**
5	293.0990	pos	13	20852	**7.3**	5	25052	11.0	0	30153	5.1	**8.1**
6	822.3179	pos	0	6037	**5.6**	0	5936	15.6	0	5073	10.7	**13.8**
7	961.3810	pos	10	1237	**12.0**	0	1200	23.7	0	1393	16.4	**19.9**
8	777.2214	pos	19	35874	**13.2**	7	30542	11.7	33	45969	9.9	**10.7**
9	243.9954	pos	16	118614	**10.9**	24	145338	6.6	39	120018	8.1	**7.3**
10	275.9675	pos	0	7365	**7.5**	0	8855	10.2	0	7871	8.6	**9.5**
11	327.0657	pos	37	225275	**8.1**	25	160983	16.6	66	190328	5.3	**11.5**
12	307.9395	pos	0	1681	**10.3**	7	1783	16.9	0	1590	5.9	**13.2**
13	359.0377	pos	8	272229	**5.5**	26	201873	10.9	73	205482	8.7	**9.9**
14	373.0532	pos	16	128913	**6.2**	11	101554	14.1	0	111450	8.6	**11.5**
15	391.0095	pos	0	6475	**5.9**	0	4712	12.3	0	4761	11.7	**12.0**
16	387.0324	pos	0	5270	**11.4**	0	5565	21.3	3	6175	10.8	**16.4**
17	261.0527	neg	0	1241	**15.0**	4	511	30.2	0	689	13.2	**21.1**
18	564.9494	neg	41	32323	**9.1**	44	12411	24.5	61	18776	22.9	**23.9**
19	385.8431	neg	0	514	**18.2**	0	245	20.7	7	462	23.7	**24.2**
20	954.6157	pos	92	1071535	**2.8**	28	506519	23.8	116	730548	15.1	**18.7**
				**Median compounds 1-20**	**8.6**			16.1			10.7	**12.7**
				**Median all 5891 peaks**	**15.0**			18.3			15.7	**18.3**

[1] based on 4 LC-MS blank runs (no injection) and 8 replicate injections from the "injection QC" vial (containing a pool of extracts), spread over the entire time range of the LC-MS analysis

[2] based on 3 extraction blanks and 6 replicate extractions of a pooled sample ("extraction QC") per extraction batch, spread over the entire extraction sequence

Comments: injection QC sample and extraction QC samples are based on different pools.

ROC curves of models 17 and 18

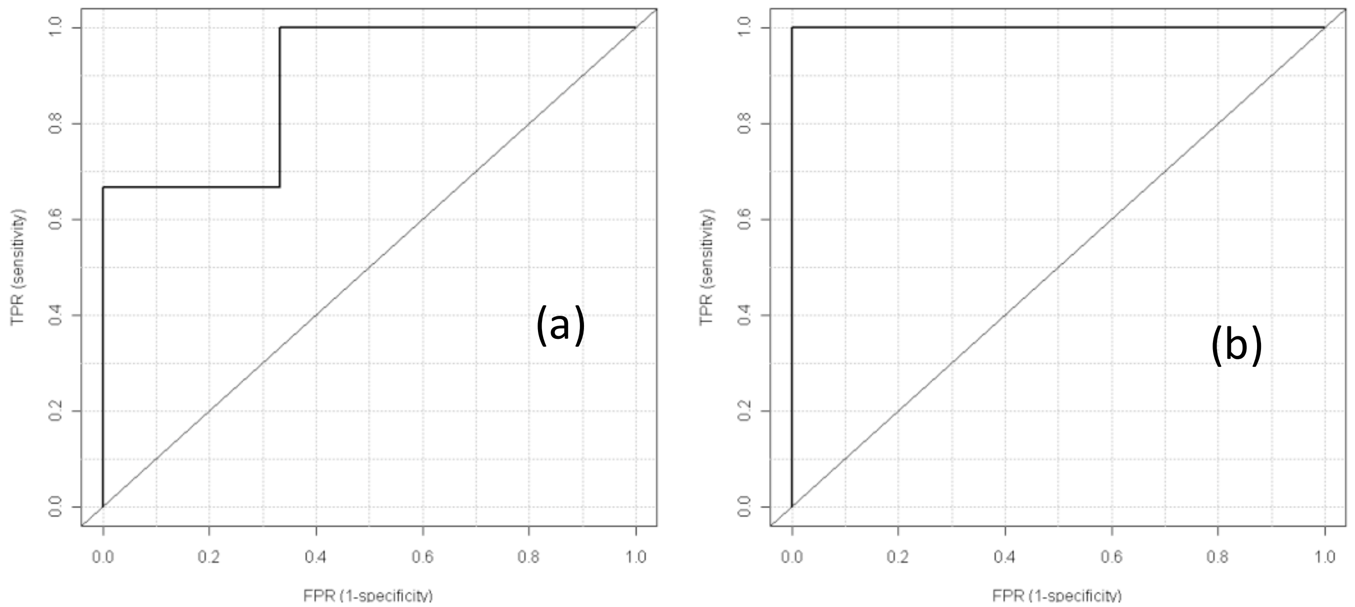

Fig. S2. ROC curves of external validation (a) model 17: AUC=0.889, p=0.048 (b) model 18: AUC=1, p=0.012. Classes: 0 – organic, 1 – conventional
